# Methyl Jasmonate: An Alternative for Improving the Quality and Health Properties of Fresh Fruits

**DOI:** 10.3390/molecules21060567

**Published:** 2016-05-31

**Authors:** Marjorie Reyes-Díaz, Tomas Lobos, Liliana Cardemil, Adriano Nunes-Nesi, Jorge Retamales, Laura Jaakola, Miren Alberdi, Alejandra Ribera-Fonseca

**Affiliations:** 1 Departamento de Ciencias Químicas y Recursos Naturales, Facultad de Ingeniería y Ciencias, Universidad de La Frontera, Casilla 54-D, Temuco 4811230, Chile; miren.alberdi@ufrontera.cl; 2 Center of Plant, Soil Interaction and Natural Resources Biotechnology, Scientific and Technological Bioresource Nucleus (BIOREN-UFRO), Universidad de La Frontera, Casilla 54-D, Temuco 4811230, Chile; alejandra.ribera@ufrontera.cl; 3 Programa de Doctorado en Ciencias de Recursos Naturales, Facultad de Ingeniería y Ciencias, Universidad de La Frontera, Temuco 4811230, Chile; tlobos@gmail.com; 4 Departamento de Biología, Facultad de Ciencias, Universidad de Chile, Casilla 356, Santiago 7750000, Chlie; lcardemi@gmail.com; 5 Max Planck PartnerGroup at Departamento de Biologia Vegetal, Universidade Federal de Viçosa, Viçosa-Minas Gerais 36570-900, Brazil; nunesnesi@ufv.br; 6 Departamento de Horticultura, Facultad de Ciencias Agrarias, Universidad de Talca, Talca 3465548, Chile; jretamal@utalca.cl; 7 Department of Arctic and Marine Biology UiT, The Arctic University of Norway, Tromsø NO-9037, Norway; laura.jaakola@uit.no; 8 Departamento de Producción Agropecuaria, Facultad de Ciencias Agropecuarias y Forestales, Universidad de La Frontera, Temuco 4811230, Chile

**Keywords:** antioxidants, fruit quality, human health, jasmonates, pre-harvest, post-harvest

## Abstract

Methyl jasmonate (MeJA) is a plant growth regulator belonging to the jasmonate family. It plays an important role as a possible airborne signaling molecule mediating intra- and inter-plant communications and modulating plant defense responses, including antioxidant systems. Most assessments of this compound have dealt with post-harvest fruit applications, demonstrating induced plant resistance against the detrimental impacts of storage (chilling injuries and pathogen attacks), enhancing secondary metabolites and antioxidant activity. On the other hand, the interactions between MeJA and other compounds or technological tools for enhancing antioxidant capacity and quality of fruits were also reviewed. The pleiotropic effects of MeJA have raisen numerous as-yet unanswered questions about its mode of action. The aim of this review was endeavored to clarify the role of MeJA on improving pre- and post-harvest fresh fruit quality and health properties. Interestingly, the influence of MeJA on human health will be also discussed.

## 1. Introduction

Several metabolic processes in plants are regulated by internal signals, such as plant hormones. One of the metabolic processes that plants must regulate is stress tolerance to be able to withstand different types of stress. In this context, phytohormones such as methyl jasmonate (MeJA), part of the jasmonate family, regulate important aspects of plant physiology [1]. These include the antioxidant systems used to ameliorate the oxidative stress induced by all kinds of biotic and abiotic stress [2]. Methyl jasmonate is involved in various plant functions from the morphological to the molecular level [3]. Given its volatile nature and ability to diffuse through biological membranes, MeJA is considered an important plant hormone that can mediate intra- and inter-plant communications, modulating plant defense responses, including antioxidant systems [4,5]. In addition, it has been shown that foliar applications of MeJA bring about changes in the gene expression responsible for fruit ripening, pollen production, foliar buds, shoots and root hair growth, as well as resistance to pest and pathogen attacks [6]. Plant response to MeJA application has been studied in various fruit crops such as *Malus domestica* (apple) and *Rubus idaeus* (raspberry) and *Fragaria chiloensis* (Chilean strawberry), among others [7,8,9].

It has been suggested that applying MeJA reduces the activity of enzymes that hydrolyze glycosidic linkages among cell wall components to induce cell wall softening in fruits, thus improving firmness and resistance to mechanical damage and indirectly reducing microbial attack [10]. Earlier findings have supported MeJA as a chemical elicitor of defense mechanisms rather than an antimicrobial itself [11]. Despite the visual benefits, there is a paucity of information regarding the effects of MeJA on the physiological processes which occur in the cell wall, *i.e.*, post-harvest rot in fruits due to microbial action, loss of firmness and mechanical damage [12]. Thus, this review endeavors to clarify the role of MeJA on improving pre- and post-harvest fresh fruit quality and health properties. Interestingly, the influence of MeJA on human health will be also discussed.

## 2. Methyl Jasmonate Biosynthesis and Signal Transduction Pathway

**Figure 1:**
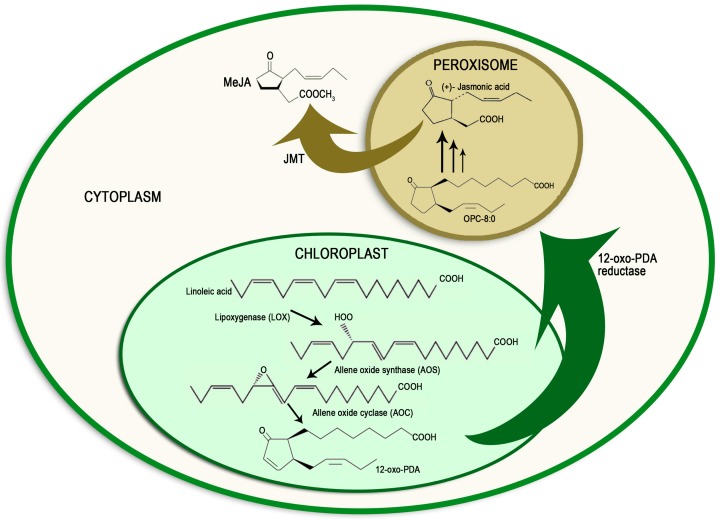
Methyl jasmonate biosynthesis involves chloroplast, cytoplasm and peroxisome. This synthesis scheme is based on studies of *Arabidopsis thaliana* plants. Abbreviations: AOC, allene oxide cyclase; AOS, allene oxide synthase; JMT, jasmonic acid methyl transferases, LOX, lipoxygenase; OPC, 3-oxo-2-(2′-pentenyl)-cyclopentane-1-octanoic acid; PDA, phytodienoic acid.

Methyl jasmonate (MeJA) is a linolenic acid (LA)-derived cyclopentanone-based compound with wide distribution in the plant kingdom [6]. It was first isolated from *Jasminum grandiflorum* (jasmine) petal extract [13]. Its chemical structure comprises a hydrocarbon ring with two functional groups: a carbonyl group (ketone) and a methyl ester group (carboxylic acid). It has two chiral carbons [6]. MeJA biosynthesis starts in the chloroplast by enzymatic oxidation of unsaturated fatty acids present in the membranes due to the lipoxygenase (LOX) that converts LA into 13-hydroperoxylinolenic acid (Figure 1). The enzymes catalyzing these reactions are allene oxide synthase and allene oxide cyclase [14]. Subsequently, 12-oxophytodienoic acid (12-oxo-PDA) is formed [14], then (−)-7-iso-JA is synthesized in the peroxisomes after three β oxidation-reduction steps (Figure 1) [4]. Afterward, MeJA is produced in the cytoplasm by reactions catalyzed by JA methyltransferases (JMT) (Figure 1) [4,15]. The detail of MeJA biosynthesis and signaling pathway are widely reported and discussed in previous reports [4,5,16,17]. However, MeJA signaling pathway is partially known. It is known that the concentration of MeJA in plants varies depending on tissue type, phenological stage and external stimuli [18]. Thus, the highest MeJA levels are reported in reproductive tissues and flowers, whereas lower levels are found in mature leaves and roots [19]. Due to its volatile nature, it has always been considered a communication molecule among plants [6]. Even though the eliciting power of MeJA has been investigated and verified by the production of secondary metabolites in many crops, the MeJA signal transduction pathway is only partially known, although many aspects are still being studied in the normal plant response to biotic or abiotic stress [4,5]. Recent research in *Cucurbita maxima* (squash) showed that the lateral exchange of phytohormones—including jasmonates—is a more appropriate mechanism for plant defense than long distance translocation [20]. It is presumed that MeJA interacts with specific receptors in membranes and the nucleus that activate a signaling pathway, resulting in the induction of transcription factors with activation or repression of MeJA-regulated genes [21]. A lack of knowledge about specific MeJA activity makes the study of receptors more difficult; therefore, the signal transduction pathway has been discovered through analysis of mutants [6,22]. The COI1 (coronatine-insensitive1) protein was discovered through research into the *coi1* mutant of *Arabidopsis* *thaliana* and *Str* (strictosidine synthase) gene present in *Catharanthus roseus* (vinca), among others, and is involved in the jasmonate signaling pathway and participates in such activities as pollen development and disease defense. Nonetheless, jasmonate-specific targets still need identification [16,22]. Recent findings in *A. thaliana*, *Solanum lycopersicum* (tomato) and *Nicotiana tabacum* (tobacco) showed that the *Coi1* gene encodes the F-box component of a SKIP–CULLIN–F-box (SCF) complex, involved in the ubiquitination of the JAZ (Jasmonate ZIM-domain) proteins [22]. The COI1 F-box confers specificity on the substrate recognizing the JAZ proteins, which are targets of the proteasome for degradation in the presence of the hormone. The JAZ proteins are repressors of the JA-induced transcriptional activity, and when these proteins are degraded, gene expression is induced. The *Coi1* encoding by the F-box is required in almost all JA-dependent responses; this box recognizes JAZ proteins, which repress JA-induced transcriptional activity. This F-box was discovered by the *coi1* mutant of *A. thaliana*.

**Figure 2:**
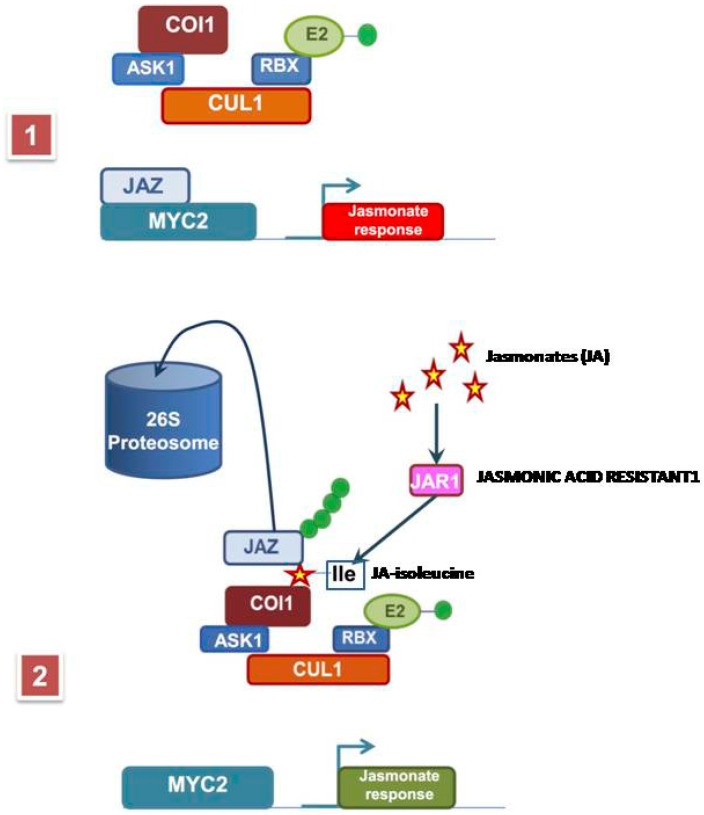
The F-box protein receptor for isoleucine jasmonate. (**1**) shows the F-box and the gene to be transcribed before jasmonate application; (**2**) shows the F-box binding the isoleucine jasmonate, the withdrawal of the JAZ protein from the promoter, the ubiquitination of the JAZ protein to be degraded in the proteosome, and the gene promoter free of the JAZ and ready to be transcribed by MYC2. ASK1, Apoptosis Signal-regulating Kinase 1; COI1, *Arabidopsis* coronatine-insensitive1; CUL1, component of the SCF complex; JAR1, Jasmonic Acid-Resistant 1; JAZ, Jasmonate ZIM-domainis a transcriptional regulator protein; MYC2, transcription factor; RBX, component of the SCF complex.

COI1 is one of the F-box proteins and a co-receptor of isoleucinejasmonate. Santner and Estelle [22] suggest that the COI1 protein is the site where JA perception binds the JA-isoleucine, the active form of JA was required to trigger JA responses. JA-isoleucine is synthesized by the enzyme jasmonate-amidosynthetase also named JAR1. This enzyme is a member of the GH3 family of proteins and catalyzes the formation of a biologically active jasmonyl-isoleucine (JA-Ile) conjugate. Also, this conjugate is considered a plant hormone today [23]. Together with the other F-box proteins (ASK1, RBX1 and CUL1) this protein makes the E2 ubiquitin ligase that takes the JAZ protein and ubiquitinates it to be sent to the proteasome to be degraded. Once JAZ proteins are removed from the promoter, the binding of the transcription factor MYC2 allow the transcription of the gene (Figure 2).

When the SCF binds, the JA-isoleucine immediately binds the JAZ protein, thereby producing the derepression of MYC2-dependenttranscription of jasmonate-responsive genes (Figure 2) [22]. However, despite the discovery of these proteins, many issues regarding the MeJA signaling pathway still remain unknown.

Interestingly, it has been reported that the MeJA signaling pathway may mediate the light induction of plant development. As response to light, phytochrome and cryptochrome induce transduction signals to influence the jasmonate signaling pathway triggering defense mechanisms and developmental responses in plants. Research has been conducted to reveal new mechanistic insights into how plants might integrate light and jasmonate signals to modify plant growth and development, and defense against pathogens and pests [24].

Far red (FR) light appears to regulate different JA-dependent responses differentially. This regulation is performed through the JAZ proteins. For instance, the *coi1* mutant under different light regimes showed different light responses. The *coi1* mutant flowers under a long-day regime instead of a short-day regime, and flowers earlier than wild-type plants [25]. On the other hand, the *coi1* mutant shows an enhanced Shade Avoidance Syndrome (SAS) response when the seedling develops under a low R:FR ratio. The hypocotyls are 30% longer than those of the wild type. Additionally, FR light/SAS negatively regulates JA-dependent pathogen defense genes, while it positively regulates JA-dependent wound/insect defense genes. In this regulation MYC2 transcription factor is involved in the JA pathway. Therefore, the defense responses against pathogens and insect attacks induced by MeJA are modulated by the FR light demonstrating that phytochrome is also involved in the defense mechanism [26].

## 3. Pre-Harvest Responses to MeJA Applications

The pre-harvest application of MeJA to plants has several effects, depending on the crop, dosis and phenological stage. Pre-harvest treatments on *Pharbitis nil* (Japanese morning glory) produced effects similar to abscisic acid applications, reducing the growth of leaves, roots, buds and shoots; however, these effects were partly reversed by application of gibberellic acid (GA3) [27]. Sprayed on *Glycine max* (soybean) (1 mM MeJA) and *Hordeum vulgare* (barley) (0.05 mM MeJA) it affects the transpiration rate due to stomatal closure [28]. Moreover, MeJA increased anthocyanin content and superoxide dismutase (SOD), glutathione reductase (GR), catalase (CAT) and peroxidase (Px) activities to counteract the oxidative stress induced by decreased photosynthetic activity and altered chlorophyll content in *A. thaliana* [29]. After MeJA applications, the antioxidant activity also increased in *Lactuca sativa* (lettuce) [30] and *Myrica rubra* (chinese bayberry) (0.1, 1 mM). In both plant species, total phenolic content increased due to enhanced phenylalanine ammonia-lyase (PAL) activity in response to MeJA treatments [31]. Kim *et al.* [32] reported a significant increment of the total phenolic content in sweet basil after 0.1 and 0.5 mM MeJA treatments compared with the control, being rosmarinic acid and caffeic acid the strong antioxidant constituents of this species.

Otherwise, recent studies have provided evidence that phenolic compounds influence the transport or action of some hormones, modulating several developmental stages of plants. Specifically, research has given evidences that the polar transport of auxin is modulated by flavonoids. For instance, flavonoids, such as quercetin, kaempferol, and apigenin have been shown to inhibit auxin polar transport. As a consequence, auxin is accumulated in the plant [33]. All this suggests that flavonoids are integral components of the plant signaling machinery. Using genome-wide RNA accumulation, Pourcel *et al.* [34] identified the set of genes associated with stress responses, cell trafficking and cell signaling with *A. thaliana* naringenin-treated *tt5* mutant (transparent testa 5, *tt5*). For this, they used seedlings of a chalcone isomerase mutant grown under conditions of anthocyanin induction, in the presence or absence of the flavonoid intermediate naringenin, a product of the chalcone isomerase enzyme. They found that naringenin increases the flow of the flavonoid pathway, inducing jasmonate biosynthetic genes. The results suggest that *Arabidopsis* can likely sense flavonoids as a signal for multiple fundamental cell processes, including MeJA biosynthesis [34].

As mentioned above, most MeJA responses have been identified by exogenous application of several concentrations of MeJA to tomato mutants such as COI1 and JAI1 [33]. Since the postharvest period is the main research focus of MeJA applications on fruits, most of these effects have been studied during this stage [6]. Despite pre-harvest MeJA applications having been little studied, this is the stage when fruit is most receptive to agrochemical applications. It has been shown in sweet cherry, for example, that MeJA treatment in early vegetative development stages produces better post-harvest responses against the pathogenic fungi *Monilinia fructicola* (brown rot, 0.2 mM). The mode of action occurs by enhancing PAL and β-1,3-glucanase activity that inhibited mycelial growth and spore germination of this fungus [35]. Rudell *et al.* [36] also found that 0.5 mM MeJA application to apples enhanced β-carotene biosynthesis through adaptation to cold temperatures, which reduces orchard temperature fluctuations and confers photoprotection on the fruit.

Also in apples, a single spray of MeJA resulted in a great increase in red blush, export-grade fruit, accumulating phenolic compounds such as cyanidin 3-galactosides of anthocyanins, chlorogenic acid, phloridzin, flavanols and flavonols in fruit skin. The MeJA was better than other treatments without affecting fruit quality [37]. Interestingly, the expression of the gene *CYP71A2* encoding the cytochrome P450s was induced by MeJA. This cytochrome seems to be crucial for avocado fruit ripening [35]. Recently, MeJA application at preharvest stage in raspberry plants resulted in a significant increase of relevant health promoting compounds such as ellagic acid, quercetin and myricetin. The authors concluded that this increase is due to a promoting effect of MeJA on PAL enzyme activity [38]. Indeed, this conclusion was previously confirmed by Wang *et al.* [31], where an increase in the PAL activity was observed in Chinese bayberry as response to MeJA application. In this sense, Kucuker *et al.* [39] reported that MeJA-treated trees of *Prunus salicina* (plums) had higher yields and maintained significantly higher flesh firmness than controls; however, the diameters of MeJA-treated fruits were lower than the control fruits. Despite of that, the authors indicated that preharvest MeJA treatment during the ripening of plums might be considered as an efficient tool for preserving fruit flesh firmness at commercial harvest. Similar results in the same species were obtained by Martínez-Esplá *et al.* [40]. It was reported that preharvest application of MeJA also improved the fruit quality and antioxidant activity of *Prunus salicina* during postharvest storage [41]. The most effective concentration was 0.5 mM of MeJA, since both non-enzymatic and enzymatic activity were higher in treated than control plums during storage, which could account for the delay in the postharvest ripening process and the extension of shelf-life. Thus, most studies suggest that preharvest MeJA applications could be a promising tool for increasing fruit quality andextending shelf-life, but the optimum concentration of this hormone is species and cultivar-dependent.

## 4. Post-Harvest Responses to MeJA Applications

In recent years, the requirement of sustainability and food security has led to dramatic changes in fruit marketing for different target markets. This has favored the emergence of trade barriers that limit pesticide residues. In this context, there have been efforts to reduce the use of inorganic pesticides, and when these are employed the preference is to apply organic forms [42]. In this way, MeJA as a natural compound has no restrictions for post-harvest applications, and it has, therefore, been tested to improve the post-harvest life of many fruit crops [8]. The MeJA, as a phytohormone, is present in different plant organs, but the largest concentrations are found in flowers and fruits [43]. It has an important effect on the content of secondary metabolites present in different kinds of fruit and is also important for developing natural defenses against abiotic stresses and post-harvest decay [44]. Most MeJA treatments are heading towards improving fruit resistance to detrimental effects during storage, including chilling injury in fruit crops like *Mangifera indica* (mango, 0.1 mM), *Ananas comosus* L. (pineapple, 0.01 mM), and *Eriobotrya japonica* Lindl. (loquat, 0.1 mM) by reducing the increase in lipoxygenase (LOX) through a decrease in lipid insaturation present in cell membranes, as well as a decrease in ion leakage and an increase in PAL activity [12,45]. Increases in peroxidase (POD) activity, regulation of Ca content and effects on cell wall degradation were observed after MeJA applications of 0.1mM in *Prunus persica* (peach) fruits [46]. Abscisic acid (ABA) and polyamines content were also affected by exogenous MeJA treatments of 1 mM on *Cucurbita pepo* (zucchini squash) and 0.2 mM on peach. Also, spermidine and spermine levels were increased in response to MeJA treatments, producing inhibition of degradative enzymes, stabilized membrane structure and also reduced lipid peroxidation [47].

On the other hand, with the onset of fruit ripening, ethylene biosynthesis enhances the normal senescence process in climacteric fruits by increasing the respiration rate and polysaccharide solubilization, among others. Ethylene synthesis can be reduced by storing climacteric fruits at 5 °C or lower [48]. In this way, MeJA treatments (10 mM) have shown a positive response in fruits by enhancing the ethylene biosynthetic enzymes 1-aminocyclopropane-1-carboxylic acid (ACC) oxidase and ACC synthase in tomato and apple fruits, which enhances fruit pigmentation and ripening [36,49].

It has been reported that post-harvest MeJA 0.01 mM applications on loquat fruit cause a higher unsaturated/saturated fatty acid ratio, which increases resistance to chilling injury [12]. MeJA treatments on stored fruits of mango (0.1 mM), pineapple (0.01 mM), loquat (0.1 mM) and peach (0.1 mM) reduced symptoms of chilling injury when the fruits were stored at a low temperature. This detrimental effect occurs primarily due to the collapse of the cell wall by several physiological processes such as ion leakage, solubilization of polysaccharides and ethylene biosynthesis [12,45,46]. Some experiments have revealed that 0.1–10 mM of MeJA applications can induce changes in the color of apple and mango by degrading chlorophyll content and enhancing carotene accumulation by promoting ethylene biosynthesis [49,50].

The post-harvest life of fruits has always been determined by visual appearance (freshness, color and presence/absence of decay or physiological disorders), texture parameters (firmness, crispness and juiciness) and phytosanitary condition. The qualities of post-harvest fruit have brought about innovation in horticulture research in terms of crop breeding, cultural practices and post-harvest handling and storage technology. The timing of fruit shelf life and fungal infection during this timing has been the main factors that may reduce fruit quality [51]. Although the most effective approach for controlling the incidence of diseases in fruits is the use of synthetic chemical fungicides, effective and non-toxic approaches must be developed to control this problem at harvest time. For this reason, an attractive alternative to reduce the incidence of diseases in fruits is the use of natural hormones such as MeJA. It has been reported that exogenous MeJA applications enhance postharvest disease resistance in fruit, reducing fungal attack (Table 1), allowing a longer and better postharvest life [8,52]. The induction of fruit resistance during postharvest appears to be an important strategy for reducing the incidence of diseases owing to the defense mechanisms in the plant itself, which has a broad-spectrum antibacterial property [44,53].

The preventive application of MeJA at 100 µM on *Citrus reticulata* (mandarin) significantly decreased the disease incidence and inhibited the extension of the lesion diameter of the *Penicillium digitatum* (green mold) compared to the control (Table 1). However, this study indicates that the method of combining MeJA with *Cryptococcus laurentii*is effective in a way that MeJA alone is not efficient in reducing the incidence of green mold in this fruit. The authors suggested that mechanism of action induces the natural resistance of mandarins and MeJA stimulates the growth of this antagonistic yeast on the fruit surface [54].

Methyl jasmonate induced the expression of plant defense genes in loquat, *Vitis vinifera* L. × *Vitis labrusca* L. cv. “Kyoho” (grape berry), and tomato, especially chitinase and β-1,3-glucanase, both encoding pathogenesis-related (PR) proteins [57,62,65]. In this research, the genes induced by MeJA codifying for chitinase and β-1,3-glucanase were able to hydrolyze the chitin polymers of fungal cell walls, indicating that these genes are involved in the plant defense mechanisms against fungal infection. Normally, small doses of fungicides are used to control post-harvest diseases. MeJA application to sweet cherry controls the fungus *M. fructicola*. However, the MeJA vapor method is not as effective as spraying or dipping the fruit in the pesticide because of the fruit’s thick skin (Table 1) [61]. The maximum possible MeJA dose is required to control the fungus *Erysiphe necator* (powdery mildew) in grapevines due to the dense foliage present at the moment of application (Table 1) [56]. Similar results were observed for Chinese bayberry fruit, where MeJA treatment activated a series of defense responses, including oxidative burst, the accumulation of PR proteins and secondary metabolites, which resulted in enhanced disease resistance in MeJA-treated fruit infected by *Penicillium citrinum*, reducing decay incidence. The authors suggested that MeJA induces resistance in Chinese bayberry through a phenylpropanoid pathway, which results in a physical barrier [64]. These findings were confirmed recently by Wang *et al.* [57], where a low concentration of MeJA (10 μM) induced disease resistance against *Botrytis cinerea* (botrytis rot) infection and reduced disease incidence in *Vitis vinifera* (grapevine), triggering a priming defense mechanism in these fruits (Table 1) [66]. Similar results were found by Zhu and Tian [62] for tomato (Table 1). Despite all investigations conducted on the effects of MeJA on fruit diseases, further studies will be performed to elucidate the molecular mechanisms underlying the MeJA-induced defense responses in postharvest fruits.

## 5. MeJA and Its Association with Other Post-Harvest Technologies

Despite MeJA having been studied from a protective perspective, there is limited knowledge regarding its interaction with other compounds used as post-harvest treatments. These associations can induce signaling pathways that may initiate subsequent cell responses [66].Thus, one of the elements that can interact with MeJA is calcium (Ca). It is well known that Ca is a nutrient and a signal transducer that regulates the metabolism in several fruits and has a key role on the structure of cell wall. In fact, the mechanism by which increased Ca levels in tissues reduce decay and maintain firmness seems to be related to the accumulation of Ca^2+^ in the cell wall through Ca pectinates (CaP) [67]. Adequate tissue Ca^2+^ concentration maintains fruit firmness, reduces the incidence of physiological disorders, increases resistance to fungal pathogens, delays fruit ripening, and maintains fruit quality for a longer period [68]. Expression of ZmCPK11 (calcium-dependent protein kinases), a member of the *Zea mays*(maize) Ca-dependent protein kinases (CDPKs) family, is induced by applying MeJA and mechanical wounding with a rapid increase in the activity of a 56-kDa enzyme, demonstrating that Ca^2+^ is the signal transducer of this enzyme activity. Methyl jasmonate probably mediates both the expression of the *ZmCPK11* gene and the presence of Ca^2+^ in the cytoplasm to activate the kinases [69]. Other evidence suggests that the MeJA signaling process indeed changes the concentration of free Ca^2+^ in the cytosol [66]. However, to date no studies have reported MeJA as an inducer of cell wall CaP formation to supply firmness to the cell wall through Ca impregnation of the cell wall. This MeJA role remains to be investigated.

Another volatile compound used in post-harvest technology is ethanol (ETOH), produced by some fruits under anaerobic conditions. It accumulates rapidly in anaerobically-stored fruits without affecting their quality [70]. Exogenous application of ETOH (2 mL·kg^−1^ fruit) vapor inhibited ethylene biosynthesis, regulating tomato fruit ripening without reducing fruit quality [70]. ETOH 35%–50% treatments have post-harvest antimicrobial attributes, dipping eliminated bacterial and fungal agents, as well as enhanced organoleptic quality and reduced table grape decay [71]. Strawberries treated with 0.1mM MeJA in conjunction with ETOH showed higher antioxidant capacity, total phenolics and anthocyanins than those treated with ethanol or an untreated control [55].

Exposure to UV-C radiation during pre-storage of peaches reduced chilling injury and decreased fungal decay; fruit firmness was also increased and ripening was delayed, although ethylene production was stimulated [72]. Higher accumulation of secondary metabolites such as putrescine, spermidine and spermine was also found after UV exposure ofmangoes [73]. Higher accumulation of polyamines in response to UV-C radiation might be helped by increasing the resistance of fruit tissue to deterioration and chilling injury [72]. Grapefruit, mangoes and zucchini squash treated with UV-C had greater PAL activity, and lower fungal and microbial development due to enhanced biosynthesis of antioxidant compounds such as phenolic acid and flavonoids. Total soluble solids (TSS) and titratable acidity (TA) were not affected, and fruit quality attributes were maintained [50]. A combination of post-harvest treatments with MeJA could extend the shelf life of fruits by enhancing the antioxidant activity and polyamine content. Thus, ethylene synthesis must be studied in depth for being able to decrease its content and thereby extend post-harvest life.

## 6. JAs and MeJA as Health Molecules

Jasmonates and their derivatives can exhibit both indirect and direct effects on human health. In the first way, it has been reported that pre- and post-harvest MeJA applications can induce the synthesis of natural products with healthy properties in some plant species, then improving their beneficial on human health.

Fruits are recognized as important sources of vitamins, minerals, and depending on the fruit crop, of antioxidant compounds such of phenolic origin such as anthocyanins, flavonoids and phenolic acids. Therefore, some fruits have shown high radical scavenging activity, thus making them effective at inhibiting oxidation of human low-density lipoproteins. Several epidemiological studies show that human diets rich in fruits [74] and natural polyphenols synthetized by plants [75] can reduce the risk of chronic and degenerative diseases, such as cancer. Currently, there are a number of UV-protective compounds that provide high UV-B solar protection for humans [76] and these are of interest in the search for natural photoprotective compounds from several organisms, including plants [77]. More specifically, anthocyanins are probably the largest group of phenols in the human diet, which have been used for several therapeutic purposes, including the treatment of diabetic retinopathy, fibrocystic disease, and vision disorders [78,79]. In addition, anthocyanins can serve as radiation-protective, vasotonic, and chemoprotective agents [80], thus decreasing the fragility of capillaries, inhibit blood platelet aggregation, and strengthen the collagen matrix of connective tissues [81]. The healthy properties of fruits are affected by several factors such as genetic background, environmental conditions, cultural practices and post-harvest handling. In this way, as mentioned above, pre- and post-harvest MeJA applications can induce the synthesis of natural products in some plant species improving their beneficial properties on human health. Recent research has shown that MeJA treatments enhance the antioxidant activity by increasing bioactive compounds in pomegranates [82] and blackberries [83], promoting their properties beneficial to human health. The application of MeJA on several fruit crops via vapor, dipping or spraying increases the concentrations of antioxidant compounds such as anthocyanins and other phenolic metabolites (Table 2), and increases antioxidant activity due to enhanced activity of antioxidant enzymes such as superoxide dismutase (SOD) [12], catalase (CAT) [84], ascorbate peroxidase (APX) [12], polyphenol oxidase (PPO) [84], PAL [38], flavanone 3β-hydroxylase (FHT) [38], 1-aminocyclo-propane-1-carboxylic acid synthase (ACS) [85], among others (Table 2). In this sense, Asghari and Hasanlooe [84] reported that MeJA applied to strawberry fruit has a good potential to be used enhancing fruit defense systems such as antioxidant enzymes (CAT, POD and PPO), increasing fruit postharvest life. Recently, Yu *et al.* [86] indicated that MeJA-treated peach fruit increase sucrose levels during cold storage. This was associated with higher sucrose phosphate synthase (SPS) and lower acid invertase (AI) levels, enhancing chilling tolerance of fruit.

On the other hand, studies have also shown that MeJA application also enhances anthocyanin accumulation in soybean seedling [89], peach shoots [90], apple fruit [91], pomegranates [82], blueberry [87], grapes [88], and strawberry [92] (Table 2). An increase in anthocyanin content has been detected in fruits after MeJA treatments despite the different doses and application method used. In this way, vapor treatment with MeJA may increase strawberry shelf life, quality and the synthesis of secondary metabolites such as phenols, without changing fruit color [55]. Unlike, although MeJA enhanced the production of antioxidants in raspberry, mainly anthocyanins, it could not decelerate the ripening process of this highly perishable fruit [8]. Post-harvest dipping treatments are most commonly used in apples in which MeJA treatments have increased anthocyanin concentrations (Table 2). In the same studies, fruit pigmentation has also been enhanced by a boost in β-carotene synthesis after chlorophyll began its degradation [7,37]. Moreover, MeJA is also able to stimulate accumulation of stilbene in leaves and berries of grapevine plants [93].

Besides plants and/or fruits rich on polyphenols, cruciferous species have been recognized as natural protectants against human cancer [94,95]. Several studies have reported that the anticarcinogenic activity of Brassicaceae species is attributed to the glucosinolates (the largest secondary metabolites of these species) and their breakdown products. Kassie *et al.* [96] was the first report that juices from a series of Brassicaceae species were antimutagenic in the Ames test. Later, this result was verified for broccoli by Martínez *et al.* [97] and Baasanjav-Gerber *et al.* [98]. More recently, research has revealed inverse associations between the intake of cruciferous vegetables and lung cancer in non-smoking women [94], gastric cancer [95], and colo-rectal cancer [99]. However, it has been reported that some glucosinolates and their breakdown-products can have mutagenic activity [100]. Pieterse and Dicke [101] reported thatglucosinolate biosynthesis can be affected via signaling molecules (plant hormones), e.g., jasmonic acid, salicylic acid, and ethylene. Indeed, juices from steamed pakchoi (*Brassica rapa* ssp. chinensis) can be strongly mutagenic [102]. The same authors found that the presence of MeJA led to 20-fold enhanced mutagenic activity in steamed pakchoi sprouts, and pointed out that this represents a molecular mechanism associated with tumor initiation rather than chemoprevention. There is evidence indicating that some plant molecules exhibit anti-inflammatory properties, induce carcinogen detoxification (phase-ii) enzymes, and are able to modulate subcellular signaling pathways of cancer cell proliferation, apoptosis and tumor angiogenesis [103], being proposed as chemopreventive agents [104]. More specifically, it has been reported that red wine phenols consumption (400 mL daily during 2 weeks) reduced the susceptibility of plasma (until 20%) and low-density lipoprotein (LDL) to lipid peroxidation due to their antioxidant action [105]. In this study, the reduced propensity of the volunteers’ LDL to undergo lipid peroxidation was evidenced by a 46%, 72%, and 54% decreases in the content of thiobarbituric acid reactive substances (TBARS), lipid peroxides, and conjugated dienes in LDL, in conjunction with a significant prolongation of the lag phase associated to the start of LDL oxidation. Interestingly, the same authors indicated that dietary consumption of white wine resulted in increases of both plasma (around 34%) and LDL (around 41%) propensity to suffer lipid peroxidation. Likewise, it has been shown that olive oil phenols can protect against coronary heart diseases (atherosclerosis) and some types of cancers in humans, mainly by inhibiting oxidation of LDL [106].

As was mentioned above, beside its key role as signal molecule and secondary metabolites inductor in plants, jasmonates (JAs) and MeJA also have direct effects on human and/or animal health. In this way, as reviewed by Fingrut and Flescher [73], JAs and some of their synthetic derivatives, were shown to inhibit the proliferation and to induce cell death in various human and murine cancer cell lines, including breast, prostate, melanoma, lymphoblastic leukemia and lymphoma cells. In addition, JAs exhibited selective cytotoxicity towards cancer cells even when they were a part of a mixed population of leukemic and normal cells drawn from the blood of patients with chronic lymphocytic leukemia (CLL) [73,107]. These outcomes confirmed that JAs have the ability to selectively kill cancer cells while sparing normal cells. Fingrut and Flegrut [73] found that MeJA treatment resulted in inactivation of apoptosis hallmarks (*i.e.*, cells apoptosis mediating by caspase-3 and DNA condensation and fragmentation) and increased the death receptor protein tumor necrosis factor receptor 1 (TNFR1), which is related to extrinsic apoptotic signaling in cancer cells. Additionally, they studied the effect of MeJA on breast cancer cell lines obtained similar results and shown that in general, MeJA caused higher levels of cytotoxicity on human cancer cells compared to JA (87.5% of cytotoxicity in Molt-4 cells at doses of 0.5 mM). They observed that MeJA was toxic to a series of cervical cancer lines, including SiHa, CaSki and HeLa (human papillomavirus DNA and wild type p53) and C33A (negative for HPV and contains mutant p53). Moreover, the same authors proposed that the MeJA anticancerigenic action can be explained by the induction of cell death and to a less extent with cell growth inhibition, with cell death revealing features to apoptosis and necrosis. This work also revealed that the death induced by MeJA was related to changes in the levels of p53, p21, bcl-2 and bax in the different cancer cell lines. Besides, Yeruva *et al.* [108] have shown that MeJA inhibited the proliferation of prostate cell lines by triggering S-phase arrest in PC-3 cells and G0/G1 block in DU-145 cells. Due to the anticarcinogenic effect of JAs on various tumors, its ability to inhibit the metastatic process on murine metastatic melanoma cells was also demonstrated [109,110]. In this regard, Reischer *et al.* [109] found that MeJA suppressed cell motility and inhibited the development of experimental lung metastases of B16-F10 cells, also suppressing the motility of a sub-clone of these cells over-expressing P-glycoprotein and displaying drug resistance. Interestingly, they also observed that some synthetic derivatives of MeJA (such as 5,7,9,10-tetrabromo derivative) had higher cytotoxic activity (IC_50_ of 0.04 mM) than MeJA (IC_50_ of 2.6 mM). In fact, this synthetic compound prevented adhesion of B16-F10 cells and inhibited the lung metastases at a much lower dose than the natural jasmonate. In accordance with these outcomes, Flescher [111] detected that among the naturally occurring JAs, MeJA is the most active and that the synthetic methyl-4,5-didehydrojasmonate, was around 29-fold more active than MeJA. According to Willis and Chen [112], the tumor-suppressive activity of p53 derived in the inhibition of cell proliferation through cell cycle arrest and/or apoptosis. Thus, cells mutated p53 lose the ability to induce the enzymatic DNA repair, triggering an uncontrolled proliferation and malignancy [113]. In this way, several tumors consisting of mutant p53-expressing cells showed resistance to both radiation and chemotherapeutic drugs [114]. Fingrut *et al.* [110] studied the capability of MeJA to induce death in mutated p53-expressing cells by assessing two clones of B-lymphoma cells: expressing wild-type (wt) p53 and expressing mutated p53. Their outcomes indicated that both jasmonic acid (JA) and MeJA (0.25 to 3 mM) exhibited cytotoxic to both clones. Furthermore, this study revealed that MeJA induced a rapid depletion of ATP mostly by compromising oxidative phosphorylation in the mitochondria. Flescher [111] and Cohen and Flescher [115] pointed out that three mechanisms could be suggested for the anticancerigenic action of MeJA: (i) induction of severe ATP depletion in cancer cells via mitochondrial perturbation; (ii) induction of re-differentiation in human myeloid leukemia cells via mitogen-activated protein kinase activity; and (iii) induction of reactive oxygen species-mediated apoptosis in lung carcinoma cells via generation of hydrogen peroxide and pro-apoptotic proteins of the Bcl-2family. It is noteworthy that, according to Cohen and Flesher [115], the combination of MeJA with conventional chemotherapeutic drugs and the glycolysis inhibitor 2-deoxy-d-glucose (2DG), can result in improved cytotoxic effects on human cancer cells. Finally, it has also been reported that MeJA can exert behavioral effects on animal cells [116]. Animals subjected to behavioral tests commonly exhibit a characteristic feature of immobility indicating a state of helplessness, lowered mood or despair [117]. Umukoro *et al.* [116] reported that intraperitoneal doses of MeJA have antidepressant effects due to its ability to reduce the immobility period in the forced swim and tail suspension tests in mice. In line with previous studies, the same authors suggested that the antidepressant effect of MeJA seems to involve serotonergic and noradrenergic mechanisms due to the lethal effect of yohimbine in mice. Recently, Umukoro *et al.* [118] found that MeJA exhibits specific anti-offensive aggressive activity, and they proposed this as a potential suitable treatment of reactive aggression in humans. Based on the promisingantitumor effects of JAs observed in animals, this molecule has been also proved as antitumor drugs for the treatment of canine oncologies [119,120]. Thus, there is evidences indicating that MeJA resulted in the highest inhibition of cell growth (82.2%), followed by doxorubicin (positive control, 80.7%) and JA (36.5%) of canine macrophage cell line DH82 (malignant histiocytoma) [120].

## 7. Concluding Remarks and Future Perspectives

In plants, pre- and post-harvest treatments with jasmonates (JAs) and its derivatives can increase the production of secondary metabolites such as anthocyanins, flavonoids, phenolic acids, and other antioxidant molecules, enhancing the fruit quality and post-harvest life, and their human health properties. It is well recognized that MeJA has proven to be an important natural compound that inhibits post-harvest fungal diseases and extend the shelf life of fruits. The most abundant phytosanitary studies about the protective effects of MeJA in plants have been related to fungi; therefore, the potential benefits of MeJA on the control of insect attack in fruits also merit further study. On the other hand, MeJA has been also shown to interact positively with another compounds and technological tools used for enhancing antioxidant capacity and improving fruit quality such as calcium, ethanol and UV-C. Interestingly, more recent studies have shown that JAs and its derivatives can have a direct anticancerigenic action in human systems, inducing cell death in various human cancer cell lines, including breast, prostate, melanoma, lymphoblastic leukemia and lymphomacells, inducing cell death in various human cancer cell lines, including breast, prostate, melanoma, lymphoblastic leukemia and lymphomacells. Future research should be conducted regarding the application of Jas in association with other beneficial compounds so that their synergic effect could provide more healthy fruits. Moreover, identifying the genes induced and repressed in response to MeJA treatments in plant and human systems is crucial in order to dilucidate the mode of action of MeJA, which to the date is not yet fully understood.

## Figures and Tables

**Table 1:** Effect of MeJA applications on post-harvest fungal diseases of fruit.

Crop	Doses	Application Method	Fungal Species	Fungal Effect	Reference
Strawberry	0.1 mM	Vapor	*Epiphyas postvittana*	Inhibition	Ayala-Zavala *et al.* [55]
Grapevine	5 or 15 mM	Spray	*Erysiphe necator*	Inhibition	Belhadj *et al.* [56]
	0.01 mM	Vapor	*Botrytis cinerea*	Inhibition	Wang *et al.* [57]
Loquat	0.01 mM	Vapor	*Colletotrichumacutatum*	Inhibition	Cao *et al.* [12]
Papaya	0.01 mM	Vapor	*Colletotrichumgloeosporioides*	Inhibition	González-Aguilar *et al.* [44]
Peach	0.001 mM	Vapor	*Botrytis cinerea*	Inhibition	Jin *et al.* [58]
Sweet cherry	10 mM	Vapor	*Monilinia fructicola*	No effect	Tsao and Zhou [59]
Sweet cherry	0.2 mM	Spray	*Monilinia fructicola*	Inhibition	Yao and Tian [60]
Peach	0.2 mM	Vapor	*Monilinia fructicola and Penicillium expansum*	Inhibition	Yao and Tian [61]
		Dipping			
Tomato	0.1 or 10 mM	Dipping	*Botrytis cinerea*	Inhibition	Zhu and Tian [62]
Pear	0.2 mM	Vapor	*Penicillium expansum*	No effect	Zhang *et al.* [63]
Chinese bayberry	0.01 mM	Vapor	*Penicillium citrinum*	Inhibition	Wang *et al.* [64]
Mandarins	0.1 mM		*Penicillium digitatum*	Inhibition	Guo *et al.* [54]

**Table 2:** Effect of post-harvest MeJA applications on the increase of antioxidant activity in fruits.

Crop	MeJA Doses	Application Method	Enzymatic and Non-Enzymatic Antioxidants	Reference
Strawberry	0.1 mM	Vapor	Anthocyanins, phenolic acid	Ayala-Zavala *et al.* [55]
Strawberry	8 and 16 µM	Vapor	CAT, POD and polyphenol oxidase (PPO)	Asghari and Hasanlooe [84]
Raspberry	0.01 and 0.1 mM	Vapor	Flavonoids, PAL, flavanone 3β-hydroxylase (FHT) and flavonol synthase (FLS)	Flores *et al.* [38]
Raspberry	0.024 mM	Vapor	Anthocyanins	Ghasemnezhad and Javaherdashti [8]
Blackberry	0.1 mM	Spray	Anthocyanins, phenolic acid	Wang *et al.* [83]
Blueberry	0.01–0.1 mM	Vapor	Anthocyanins	Huang *et al.* [87]
Grapes	1.78 mM	Vapor	Anthocyanins, total phenols	Flores *et al.* [88]
Loquat	0.01 mM	Vapor	Superoxide dismutase (SOD), chloramphenicol acetyltransferase, ascorbate peroxidase (APX)	Cao *et al.* [12]
Pomegranates	0.01–0.1 mM	Vapor	Total phenolic and anthocyanins	Sayyari *et al.* [82]
Apple	1 mM	Dipping	Anthocyanins	Rudell*et al*. [36]
Plum	0–1 mM	Vapor	1-Aminocyclopropane-1-carboxylic acid synthase (ACS) and 1-amino-cyclopropane-1-carboxylic acid oxidase (ACO)	Khana and Singha [85]
Peach	10 µM	Vapor	Sucrose phosphate synthase (SPS)	Yu *et al.* [86]
